# A Survey of Medicine in 1908

**Published:** 1909-01-02

**Authors:** 


### The Hospital

A Journal of

The Medical Sciences and Hospital Administration.

New Series. No. 96. Vol. IV. [No. 1163, Vol. XLV.] SATURDAY, JANUARY 2, 1909.

### A SURVEY OF MEDICINE IN 1908.

So much that is fresh is constantly being brought before the medical practitioner that it would be impossible to enter fully into the advances that occur in any single year. Many things that have been begun in one year are not completed until the next or a later year, so that they cannot be claimed as the product of any one year. Nineteen hundred and eight has seen some remarkable cases arising under the various older and more recent Acts dealing with employers' liability and workmen's compensation. The extension of these Acts has introduced a far wider and more medical, as distinct from purely surgical aspect into the questions involved. Although the newest of these Acts has now been in force for over a year there is still a good deal of uncertainty as to how it will be interpreted in particular cases. Expert knowledge upon the diseases that special trades are liable to lead to has become essential, and interesting books, papers, and discussions upon trade diseases have sprung up to meet the demand. The Poisons and Pharmacy Bill has successfully passed both Houses of Parliament and has received the Royal Assent. It touches pharmaceutical chemists more nearly than it does doctors, but there are several points in it that deal with subjects of interest to medical men. These we have dealt with elsewhere already.

The Charter of the British Medical Association is about to come before the Privy Council, and we wish it every success there. When one has to deal with any large body of men such as the British Medical Association there is probably no single point whatever upon which, if a vote were taken, there would not be a minority as well as a majority. In every collection of gentlemen, however, the minority staunchly supports the majority when concerted action by the whole body is required in public view. For the minority to begin quarrelling at the last moment over resolutions that have been duly passed after long and careful consideration is unseemly, and we trust that such incipient bickering will subside spontaneously and at once.

The Royal Society of Medicine has completed its first year since its origin from the voluntary amalgamation of fourteen separate societies. The achievement is already great; the fruits promise to be greater still. Hundreds of persons who could not afford to join all the different societies they would have liked to are now able to join the equivalent sections at very little cost. Papers read before the Society are not delayed long in publication, for there is an issue of printed proceedings monthly. The first general debate was upon heredity as a factor in disease, and the discussion had to be adjourned several times, whilst on each occasion there was a packed audience representative of every branch of the profession.

The country has begun to awake to the dangers of the present system of milk suppiy in cities. The London County Council has just completed its first year of special inspection of samples of milk as they arrive at the London railway termini. We have already noticed the inspector's report: nearly one quarter of the samples taken at random contained tubercle bacilli. However, the beginning having been made, the proportion of cases in which tubercle bacilli occur should now rapidly diminish, for the consignments tested can be traced to the farms, and the offending cows can be found and eliminated from the herds. This is a tremendous advance in the right direction. The year has continued to throw much light upon the mode of origin of many cases of typhoid fever that have hitherto baffled attempts to trace them to any of the better known causes. Much has been discovered in connection with so-called "typhoid carriers"—people who have recovered completely from typhoid fever and who yet continue to excrete living typhoid bacilli in their stools for as long as even twenty or thirty years afterwards. The extreme importance of this possibility has become very obvious, and the year has seen a considerable number of cases of the kind recorded.

Of new diagnostic methods, the two which have probably been most prominent in 1908 are Calmette's and von Pirquet's tuberculo-reactions. We have discussed them both more than once. There has been a controversy as to who discovered the tuberculo-ophthalmic reaction—Wolff-Eisner or Calmette. The former makes out a good case for his priority, but the name of the latter has already become too familiar in association with the test to be quickly exchanged for the former. One thing has become very clear about the reaction, and that is that it is by no means so absolutely free from danger to the eye as was at first supposed. Doubtless the percentage of cases in which serious results ensue is small; nevertheless, the year has seen the publication of a number of untoward effects. From the point of view of treatment, the chief feature of the year has been the increasing popularity of vaccines, not only for tuberculous or staphylococcal lesions, but also for disorders produced by the gonococcus, the bacillus coli communis, the bacillus proteus vulgaris, pneumococcus, and so on. The importance of opsonic index determinations as a *sine quâ non* in vaccine treatment seems to be dwindling, but vaccines are being advocated and employed—sometimes successfully—for many different conditions.

Researches in tropical medicine already hold a prominent position in British medicine. They are beginning to exert a direct influence upon practice at home. No better example of this can be given, we think, than the discovery of the value of concentrated arsenic in organic combination such as atoxyl, soamin, and the like in the treatment of sleeping sickness, and its subsequent introduction as an adjuvant in the cure of syphilis. We look forward to a continuous exchange of ideas in the future between those who practise in the colonies and abroad and those who practise in Great Britain.

